# Cavernous sinus hemangiomas: stereotactic radiosurgery as gold standard, with robotics and AI on the horizon

**DOI:** 10.1097/MS9.0000000000004000

**Published:** 2025-09-30

**Authors:** Samia Arif, Wareesha Azmat, Maliha Khalid, Muhammad Talha, Aminath Waafira

**Affiliations:** a Department of Medicine, King Edward Medical University, Lahore, Pakistan; b Department of Medicine, Fatima Jinnah Medical University, Lahore, Pakistan; c Department of Medicine, Jinnah Sindh Medical University, Karachi, Pakistan; d School of Medicine, The Maldives National University, Malé, Maldives

**Keywords:** artificial intelligence, cavernous sinus hemangioma, robotic neurosurgery, stereotactic radiosurgery

## Abstract

Cavernous sinus hemangiomas (CSHs) are rare, highly vascular tumors of the skull base where conventional surgery carries substantial morbidity. Stereotactic radiosurgery (SRS) has emerged as the gold standard, offering >90% local control and substantial tumor shrinkage with minimal cranial nerve deficits. Hypofractionated and staged approaches further extend safety in complex cases. Robotics and artificial intelligence (AI) represent promising adjuncts, with robotics improving surgical access in select cases and AI models supporting intraoperative accuracy and outcome prediction. However, adoption is limited by technical constraints, sparse clinical evidence, and lack of validated AI algorithms. While SRS remains the primary treatment, future integration of robotics and AI requires multicenter validation, standardized reporting, and technological refinement to ensure safe translation into clinical practice.

*To the editor,*

Cavernous sinus hemangiomas (CSHs) are rare, benign vascular tumors of the skull base, arising from abnormal proliferation of thin-walled vascular channels that predispose to progressive growth and compression of adjacent cranial nerves and the internal carotid artery. Because of their highly vascular nature, conventional surgical resection carries significant risks of morbidity and mortality. Advances in stereotactic radiosurgery (SRS) have provided an effective and minimally invasive alternative; however, challenges remain regarding patient selection, dosing schemes, and long-term outcomes^[1]^. In this context, robotic-assisted microsurgical approaches and artificial intelligence-based outcome prediction models offer new possibilities to improve safety.

SRS has been the gold standard of CSH management. Gamma Knife and linear accelerator series show reliable results, with mean tumor-volume reductions of 70–88% within the first year, and a near 90% reduction after 12 months reported by some studies. Local control rates exceed 90% while new cranial-nerve deficits occur in fewer than 10% of cases. For larger tumors or those surrounding the internal carotid artery, hypofractionated, or staged protocols provide a high degree of safety and durable efficacy. These results establish SRS as an effective standard of care, underscoring the narrow margin for improvement^[2]^. However, large tumors or extensive vascular involvement may need adjunctive approaches to SRS.

Recent studies have considered robotic-assisted surgery and artificial intelligence as adjuncts to radiosurgical management of CSHs. A systematic review of 22 clinical and cadaveric studies reported that robotic platforms improved surgical access, three-dimensional visualization, and instrument dexterity in challenging skull-base regions. These results indicate that robotics can be of benefit in cases where SRS is contraindicated or poorly tolerated^[3]^. Moreover, artificial intelligence (AI) is emerging as a valuable adjunct in robotic surgery, particularly for enhancing intraoperative accuracy. AI models may help with intraoperative accuracy, surgical margin detection, and automated skill assessment, improving precision and safety^[4]^.

Nevertheless, there are notable limitations that continue to hinder the broader adoption of current robotic systems. Existing robotic tools are bulky, with limited triangulation and no haptic feedback. Evidence is sparse, limited to cadaveric experiments and small series, limiting generalizability. Recent reviews of the anterior skull base report limited adoption attributed to technical challenges. Similarly, AI tools often lack external validation, and their applicability to neurosurgical practice has not been demonstrated. Considering these limitations, SRS should remain the primary treatment, whereas robotics should be used only for selected cases until supported by larger clinical studies and technological advances^[5]^.

In conclusion, SRS remains the gold standard for managing CSHs. Robotics and AI can be used as adjuncts, but their integration requires multicenter trials, validated algorithms, and further technological development. Adoption, however, must remain evidence-based until such advances are achieved. Collaborative registries and standardized reporting will be important in the provision of the evidence needed to support safe integration of the technologies. This letter to editor is in compliance with the TITAN guideline^[6]^.

## Data Availability

No data were generated for this manuscript.

## Research registration unique identifying number (UIN)

Name of the registry: N/A; unique Identifying number or registration ID: N/A; hyperlink to your specific registration (must be publicly accessible and will be checked): N/A.
